# Cerebral hemodynamic and systemic physiological changes in trained freedivers completing sled-assisted dives to two different depths

**DOI:** 10.1152/ajpregu.00085.2024

**Published:** 2024-09-06

**Authors:** Eva-Maria S. Bønnelycke, Tommaso A. Giacon, Gerardo Bosco, Jana M. Kainerstorfer, Matteo Paganini, Alexander Ruesch, Jingyi Wu, J. Chris McKnight

**Affiliations:** ^1^Sea Mammal Research Unit, Scottish Oceans Institute, University of St. Andrews, St. Andrews, Scotland, United Kingdom; ^2^Laboratory of Environmental and Respiratory Physiology, Department of Biomedical Sciences, University of Padova, Padova, Italy; ^3^Institute of Anesthesia and Intensive Care, Padova University Hospital, Department of Medicine (DIMED), University of Padova, Padova, Italy; ^4^Department of Biomedical Engineering, Carnegie Mellon University, Pittsburgh, Pennsylvania, United States; ^5^Neuroscience Institute, Carnegie Mellon University, Pittsburgh, Pennsylvania, United States

**Keywords:** arterial blood gas, breath-hold diving, cerebral hemodynamics, heart rate, near-infrared spectroscopy

## Abstract

Although existing literature covers significant detail on the physiology of human freediving, the lack of standardized protocols has hindered comparisons due to confounding variables such as exercise and depth. By accounting for these variables, direct depth-dependent impacts on cardiovascular and blood oxygen regulation can be investigated. In this study, depth-dependent effects on *1*) cerebral hemodynamic and oxygenation changes, *2*) arterial oxygen saturation ($\text{S}{\text{p}}_{{\text{O}}_{2}}$), and *3*) heart rate during breath-hold diving without confounding effects of exercise were investigated. Six freedivers (51.0 ± 12.6 yr; means ± SD), instrumented with continuous-wave near-infrared spectroscopy for monitoring cerebral hemodynamic and oxygenation measurements, heart rate, and $\text{S}{\text{p}}_{{\text{O}}_{2}}$, performed sled-assisted breath-hold dives to 15 m and 42 m. Arterial blood gas tensions were validated through cross-sectional periodic blood sampling. Cerebral hemodynamic changes were characteristic of breath-hold diving, with changes during ascent from both depths likely driven by decreasing $\text{S}{\text{p}}_{{\text{O}}_{2}}$ due to lung expansion. Although $\text{S}{\text{p}}_{{\text{O}}_{2}}$ was significantly lower following 42-m dives [*t*(5) = −4.183, *P* < 0.05], mean cerebral arterial-venous blood oxygen saturation remained at 74% following dives to both depths. Cerebral oxygenation during ascent from 42 m may have been maintained through increased arterial delivery. Heart rate was variable with no significant difference in minimum heart rate between both depths [*t*(5) = −1.017, *P* > 0.05]. This study presents a standardized methodology, which could provide a basis for future research on human freediving physiology and uncover ways in which freedivers can reduce potential risks of the sport.

**NEW & NOTEWORTHY** We present a standardized methodology in which trained breath-hold divers instrumented with wearable near-infrared spectroscopy (NIRS) technology and a cannula for arterial blood sampling completed sled-assisted dives to two different dive depths to account for the confounding factors of exercise and depth during breath-hold diving. In our investigation, we highlight the utility of wearable NIRS systems for continuous hemodynamic and oxygenation monitoring to investigate the impacts of hydrostatic pressure on cardiovascular and blood oxygen regulation.

## INTRODUCTION

The physiological changes experienced by breath-hold diving humans close to, or in thermoneutral conditions (diving either in a wetsuit or thermoneutral temperature water), are primarily influenced by dive duration and depth (1). Increasing dive duration increases the risk of hypoxia by extending the amount of time without access to ambient oxygen. Depth not only affects dive duration but also introduces intrathoracic pressure changes (i.e., lung compression and expansion) due to changing barometric pressure (1, 2). The effect of lung compression and expansion on arterial blood gas tensions during freediving to depth has been validated through arterial blood gas sampling in laboratory, pool, and open-water settings (2–6). This phenomenon causes a decrease in blood oxygen levels supplying the brain during ascent (1–3, 6, 7). However, it is not known whether there may be other physiological mechanisms contributing to ascent hypoxia, such as cerebral hemodynamic mechanisms engendered from barometric pressure changes affecting brain oxygenation during ascent from depth. If another hemodynamic mechanism is contributing to cerebral deoxygenation during ascent, then freedivers may be able to introduce new practices to ameliorate its effect.

Depth-related changes in heart rate occur as part of the physiological response to submersion under water (i.e., the dive response) and have also been attributed to the level of exertion during different stages of diving: actively swimming versus passive descent or ascent (8, 9). Previous studies report contradictory findings on whether exercise tachycardia overrides the cardiovascular dive response or vice versa (10, 11). To our knowledge, the influence of depth-induced intrathoracic pressure changes on heart rate in the absence of exercise remains uncertain. To disentangle the major depth-dependent influences on cardiovascular and cerebrovascular regulation, continuous local hemodynamic measurements in the brain, as well as continuous systemic measurements of arterial blood oxygen saturation delivered to the brain, and heart rate, are required.

Fujimoto and Sano and McKnight et al. have previously demonstrated the efficacy of near-infrared spectroscopy (NIRS) technology in providing continuous hemodynamic measurements noninvasively from human freedivers (12, 13). NIRS is an optical imaging method that relies on the relative absorption of near-infrared light through biological tissue (14). NIRS systems emit specified wavelengths of near-infrared light, which are differentially absorbed by chromophores within biological tissue: oxygenated hemoglobin, deoxygenated hemoglobin, and oxidized cytochrome *c* oxidase. Light scatters through tissue is attenuated by the chromophores, and then is detected by receivers of the NIRS system. The relative loss in light intensity is translated into relative changes in concentration of the chromophores. Continuous-wave NIRS systems (CW-NIRS) solely measure the abovementioned loss of light intensity, meaning relative concentration changes of the targeted chromophores are obtained as opposed to absolute concentration measurements, which can be obtained from time-resolved NIRS techniques (14). Nonetheless, CW-NIRS systems have demonstrated great value in monitoring cerebral oxygenation dynamics in real time, and the changes in concentration of oxygenated ([ΔO_2_Hb]) and deoxygenated hemoglobin ([ΔHHb]) can additionally provide measurements of heart rate and arterial oxygen saturation ($\text{S}{\text{p}}_{{\text{O}}_{2}}$) (15–18).

Although NIRS and arterial blood gas analyses have been used independently in previous studies on human freediving (2, 4–6, 12, 13, 19–21), the lack of a standardized protocol has hindered comparisons due to confounding variables such as freediving experience of participant divers, level of exercise, dry versus wet conditions, and differences in dive depth. As such, the aims of the current study were to investigate the depth-dependent effects on *1*) cerebral hemodynamic and oxygenation changes, *2*) arterial blood oxygen saturation ($\text{S}{\text{p}}_{{\text{O}}_{2}}$), and *3*) heart rate during breath-hold diving without confounding effects of exercise.

In this study, freedivers with varying levels of experience were instrumented with a CW-NIRS device for monitoring continuous changes in cerebral blood oxygenation, heart rate, and $\text{S}{\text{p}}_{{\text{O}}_{2}}$_._ Freedivers were also cannulated for arterial blood sampling at various points throughout the dive protocol. Importantly, we applied a newly developed $\text{S}{\text{p}}_{{\text{O}}_{2}}$ extraction algorithm (18) combined with the concomitant arterial blood sample measurements of ground truth arterial oxygen saturation ($\text{S}{\text{a}}_{{\text{O}}_{2}}$) to validate the accuracy of our $\text{S}{\text{p}}_{{\text{O}}_{2}}$ estimates. The dive protocol consisted of two sled-assisted dives; one to 15 m and one to 42 m of depth. By using sled-assisted dives to two separate depths we focused our investigation on depth-dependent physiological changes that occur independent of physical exercise. We highlight the utility of NIRS for continuous hemodynamic monitoring to investigate drivers of cerebral oxygenation changes during freediving. By obtaining ground truth arterial blood gas values, we validated extracted $\text{S}{\text{p}}_{{\text{O}}_{2}}$ measurements and the interpretation of changes observed in NIRS-derived hemodynamic signals. Our methodology provides a basis for future research on human freediving physiology, which could uncover ways in which freedivers can reduce potential risks of the sport.

## MATERIALS AND METHODS

### Ethics Statement

The experimental protocol adhered to the principles of the Declaration of Helsinki and was approved by the Human Ethical Committee of the Department of Biomedical Science at the University of Padova (No. HEC-DSB/03-18). Informed written consent was provided by all experimental participants before the start of the study. All procedures were conducted with measures in place to ensure the safety of the divers. All participant divers were confident in freediving at the predetermined experimental depths and could interrupt the experimental procedures at any time. In-water safety was provided by breath-hold diving instructors and by medical doctors SCUBA diving at depth. All of the medical procedures were executed by trained anesthesiologists and emergency medicine physicians.

### Near-Infrared Spectroscopy System

The waterproofed NIRS device used for the following experimental protocol, called the “PortaDiver,” was developed from an existing CW-NIRS system used in humans (PortaLite mini, Artinis Medical Systems BV, Einsteinweg, The Netherlands). The PortaDiver is a wearable, dual-length, and spatially resolved NIRS system that offers optical penetration to three separate tissue depths: 30 mm (842 nm and 761 nm optode), 35 mm (841 nm and 759 nm optode), and 40 mm (841 nm and 758 nm optode). During the experimental trials, changes in light intensity were measured at a sampling frequency of 10 Hz. These changes in received light intensity can be translated into measurements of relative concentration changes in oxygenated and deoxygenated hemoglobin ([ΔO_2_Hb] and [ΔHHb], respectively) using the modified Beer–Lambert law (22, 23), which describes changes in tissue optical absorption, assuming constant scattering. This conversion was automatically completed within the NIRS control software; “Oxysoft” (v. 3.2.70 Artinis Medical Systems BV, Einsteinweg, The Netherlands). In addition, the PortaDiver allows for the calculation of tissue oxygenation/saturation index (TSI); a proportional measure of cerebral tissue saturation with oxygenated hemoglobin (combined arterial and venous contribution). The calculation of TSI relies on the use of all three optode-detector distances to calculate a scaled absolute light absorption coefficient, yielding an estimate of the tissue’s relative concentration of oxygenated hemoglobin (24). For further detail on the development and specifications of the PortaDiver, see study by McKnight et al. (12).

### Subjects

Six trained and healthy freedivers participated in the study. The participants were mixed in gender, age, and breath-hold diving experience (see Table 1 for further information on dive participants). The number of participants was limited as this study relied on volunteer participation of experienced freedivers available at the “Y-40 The Deep Joy” facility and that were comfortable with arterial cannulation and submerged phlebotomy.

**Table 1.** Sex, age, mass, height, body mass index, and years of freediving experience of all six subjects included in the analysis of the current study

Subject	Sex	Age, yr	Mass, kg	Height, m	Body Mass Index, kg/m^2^	Years of Experience
*1*	M	60	87	1.78	27.5	7
*2*	F	29	65	1.70	22.5	6
*3*	M	64	78	1.71	26.7	46
*4*	M	57	80	1.80	24.7	6
*5*	M	47	89	1.85	26.0	9
*6*	M	49	91	1.80	28.1	8
Means ± SD		51.0 ± 12.6	81.7 ± 9.6	1.77 ± 0.06	25.9 ± 2.05	13.7 ± 15.9

The means ± SD are presented for each numerical category. For further anthropometric information on the subjects’ diving history and training intensity, see Supplemental Table S1.

### Breath-Hold Diving Protocol

**Figure 1.**
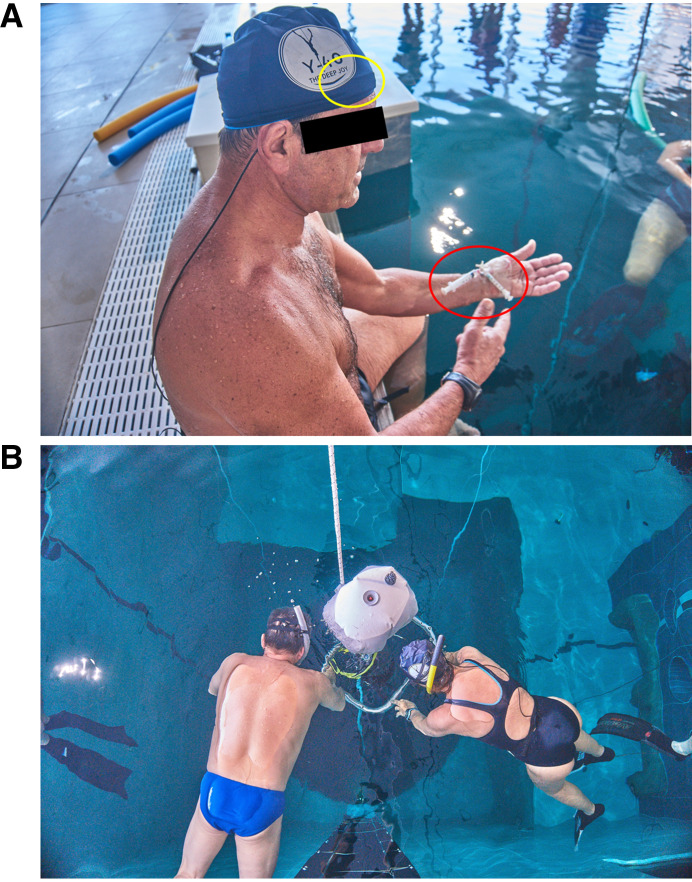
*A*: the location of the PortaDiver sensor head under the freediver’s swimming cap (yellow circle) and the arterial cannula with the three-way stopcock system for blood sampling (red circle). *B:* an instrumented freediver and their instructor holding on to the sled used for descent and ascent in the dive trials.

Participant freedivers received thorough instruction on the experimental protocol to ensure their safety and correct execution of the diving protocol. The breath-hold diving trials were conducted in the 42-m deep, indoor thermal pool of the “Y-40 The Deep Joy” facility in Montegrotto Terme, Padova, Italy, between June 2021 and December 2021. The thermal water temperature remained steady between 32°C and 34°C, within the thermoneutral zone for humans. Divers were equipped with the PortaDiver and an arterial cannula before completing the breath-hold diving trials. The PortaDiver sensor head was placed above one eyebrow covering the prefrontal cortex under the divers’ double-layered silicon swimming cap to reduce movement and light interference (Fig. 1*A*). The PortaDiver sensor body, containing the battery and control unit, was secured around the divers’ waists with a belt.

Before starting the breath-hold diving protocol, freedivers completed short warm-up dives for ∼30–60 min, at a maximum depth of 15 m, guided by their breath-hold diving instructor. These warm-up dives were not standardized to ensure the safety of the divers and give them their individually required time and routine to mentally and physically prepare for the experimental dive trials. Once the divers felt ready to begin, they were equipped with the PortaDiver and asked to complete their normal breathing preparation. The predive breathing preparation occurred over 2–5 min and consisted of controlled breathing with 8–9 breaths/min (inspiratory-to-expiratory length ratio of 1:2) and a final breath reaching total lung capacity. Divers were instructed not to hyperventilate or perform glossopharyngeal insufflation (“lung packing”). Breath-hold diving instructors were present in the water and ensured the correct execution of the breathing preparation.

For each dive trial, the divers completed a 15-m dive, then recovered at the surface for around 2–10 min before completing a 42-m dive. The recovery time between dives was determined by the participant divers to ensure the safety of the individual divers who may require different recovery durations between consecutive dives. For each dive, the dive start and end times and the time at which the divers reached the bottom of the dive depth were recorded. The divers ascended and descended with the assistance of a sled for each trial and depth to minimize any physical exertion (Fig. 1*B*). The sled-assisted ascent and descent rate was ∼1 m/s.

### Arterial Blood Sampling and Analysis

For arterial blood sample collection, an arterial cannula was inserted in the radial artery of the nondominant forearm using the Seldinger technique under sterile conditions and local anesthesia. The cannula was fixed to the skin using an adhesive band and then connected to a circuit with a lockable three-way stopcock system for underwater blood collection (Fig. 1*A*). The arterial access and blood collection protocol are explained in full in the study by Bosco et al. (2).

**Figure 2.**
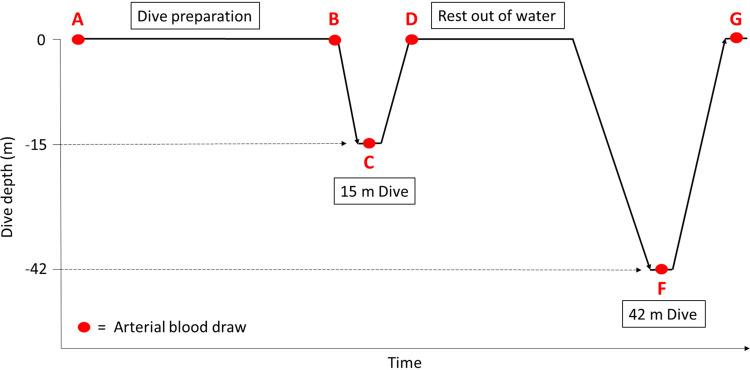
The time points at which arterial blood draws were taken during the dive protocol. To limit the amount of blood taken from participant divers, there was no pre-descent blood draw for the 42-m dive (*point E*). Therefore, *point E* was assumed to be nearly identical in terms of gas exchange to the pre-descent blood draw of the 15-m dive (*point B*).

Figure 2 visually illustrates the points at which arterial blood draws were taken during the dive protocol. The baseline arterial blood samples were taken during arterial cannulation, before the warm-up dives. The remaining blood draws were taken while the divers’ heads were submerged underwater. Therefore, after doing their breathing preparation, each diver submerged their head and extended their arm out of the water at which point a pre-descent blood draw (Fig. 2, *point B*) was taken. Then they performed their dive and at the end of ascent (Fig. 2, *points D* and *G*), the end-dive arterial blood sample was taken before they took their first breath, while their heads were still submerged. This method allowed us to consider the human body as a closed circuit reliant upon endogenous oxygen stores in terms of gas exchange. The blood samples collected at depth (Fig. 2, *points C* and *F*) were placed into a bottle that was immediately pulled up to the surface for analysis. The duration of each arterial blood draw was ∼5–10 s.

The blood samples were analyzed within 2–3 min of collection using the Stat Profile Prime Plus (Nova Biomedical Italia Srl, Lainate, Italy) blood gas analyzer. Although the blood analysis provided the full-range hematochemical values, we focused on measurements of arterial oxygen saturation ($\text{S}{\text{a}}_{{\text{O}}_{2}}$,%), partial pressure of oxygen (${\text{Pa}}_{{\text{O}}_{2}}$, mmHg), partial pressure of carbon dioxide (${\text{Pa}}_{{\text{CO}}_{2}}$, mmHg), and hemoglobin concentration (Hb, g/dL) for the purpose of this study.

### Extraction of Cerebral Hemodynamic Variables from NIRS

To provide continuous measurements of physiological changes during diving, a number of metrics were calculated using the raw [ΔHHb] and [ΔO_2_Hb] signals: *1*) changes in total hemoglobin concentration ([ΔtHb] = [ΔO_2_Hb] + [ΔHHb]), which is a proxy for relative blood volume changes; *2*) tissue saturation index (TSI), a mixed arterial and venous blood oxygen saturation; *3*) heart rate; and *4*) $\text{S}{\text{p}}_{{\text{O}}_{2}}$. For the purpose of this article, [ΔHHb], [ΔO_2_Hb], and [ΔtHb] are collectively referred to as cerebral hemodynamic variables.

To provide continuous hemodynamic measurements throughout preparation, diving, and recovery for each 15-m and 42-m dive, [ΔHHb], [ΔO_2_Hb], and [ΔtHb] measurements were extracted for 45 s before the start of the dive and 45 s after the end of the dive. Each dive depth and its analysis window (±45 s time frame) is referred to as a “dive trial” for the purpose of this article. [ΔHHb], [ΔO_2_Hb], and [ΔtHb] measurements were extracted for all three channels of the PortaDiver NIRS system, and the channel with the least noisy signal was chosen for visual inspection of the hemodynamic changes.

### Tissue Saturation Index Extraction

TSI is a proportional measure of local tissue oxygenation (also known as tissue oxygenation index), calculated using spatially resolved spectroscopy with a minimum of two wavelengths and two source-detector distances (25, 26). The PortaDiver specifications meet these criteria, and it was therefore possible to extract TSI retrospectively in MATLAB (The MathWorks, Inc., Natick, MA, RRID: SCR_001622) using the following equation (25, 26):

$$k\mu_{\text{a}}=\frac{1}{3\left(1-h\lambda \right)}{\left(\text{ln}\;10\frac{\partial \text{A(λ)}}{\partial \rho }-\frac{2}{\rho }\right)}^{2}$$

whereby a scaled absorption coefficient (*k*μ_a_) is calculated using the slope of the measured change in light attenuation (A) with the three source-detector distances (ρ) of the PortaDiver. Using this scaled absorption coefficient, the scaled absolute concentration of oxygenated (*k*[O_2_Hb]) and deoxygenated hemoglobin (*k*[HHb]) is quantified, and TSI is specified by the following equation (25, 26):

$$\text{TSI}=\;\frac{\left(k\left[{\text{O}}_{2}\text{Hb}\right]\right)}{\left(k\left[\text{tHb}\right]\right)}$$

where [tHb] is the absolute concentration of total hemoglobin ([tHb] = [O_2_Hb] + [HHb]). TSI was extracted for all dive trials included in the analysis of this article.

### Heart Rate Extraction

To provide a continuous measurement of basic cardiac function, heart rate was extracted from the NIRS measurements. Heart rate presents as regular peaks within the NIRS signal corresponding to cardiac pulsations (16). When the heart contracts during systole, arterial blood pressure increases, causing an increase in arterial blood flow volume and therefore hemoglobin concentration in tissues. The opposite occurs during diastole. The changes in hemoglobin concentration are reflected in the tissue’s light absorption and therefore detected by NIRS. Heart rate extraction was performed in MATLAB (The MathWorks, Inc., Natick, MA; RRID: SCR_001622), and the method used in this study is explained in full in the study by McKnight et al. (12). This method used the heart rate derivation algorithm presented in the study by Hakimi and Setarehdan (16). In the first stage of the extraction algorithm, a 100th-order zero-phase bandpass FIR filter between 0.1 and 4 Hz was applied to remove slow drifts and high-frequency noise in the NIRS signal. Next, the automatic multiscale-based peak detection (AMPD) method (27) was applied to detect peak points within the filtered NIRS signal and then peak-to-peak time intervals were calculated to obtain heart rate. In the final step of the derivation algorithm, a windowing approach (16) was used to reduce errors in peak detection. Heart rate was extracted for the channel with the shallowest skin-cortical tissue path for each dive trial.

### Arterial Oxygen Saturation Extraction

To provide a continuous measurement of arterial blood oxygenation, $\text{S}{\text{p}}_{{\text{O}}_{2}}$ was extracted from the NIRS measurements in MATLAB (The MathWorks, Inc., Natick, MA; RRID: SCR_001622) using a novel self-calibrated algorithm. This algorithm differs from the conventional method of $\text{S}{\text{p}}_{{\text{O}}_{2}}$ extraction (17) as it does not assume the wavelength-dependent photon pathlength to be constant; a flawed assumption as the amount of oxygenated and deoxygenated hemoglobin present in a tissue affects the pathlength of photons leading to potential spurious $\text{S}{\text{p}}_{{\text{O}}_{2}}$ estimates below 80% saturation. This is particularly important when using $\text{S}{\text{p}}_{{\text{O}}_{2}}$ estimates in freediving where divers will present $\text{S}{\text{p}}_{{\text{O}}_{2}}$ below 80% (12). There is a strong likelihood that previous freediving studies have a significant, and unaccounted for, error in their $\text{S}{\text{p}}_{{\text{O}}_{2}}$ estimates. The underlying theory of the self-calibrated algorithm and the method of its use in the current study are detailed in the study by Wu et al. (18). Wu et al. (18) used the data presented in the current study to validate the self-calibrated algorithm for $\text{S}{\text{p}}_{{\text{O}}_{2}}$ extraction. This validation relied on the ground truth arterial $\text{S}{\text{a}}_{{\text{O}}_{2}}$ measurements obtained from the arterial blood draws for calibration of the extracted continuous $\text{S}{\text{p}}_{{\text{O}}_{2}}$ measurements. The novel self-calibrated algorithm provided more accurate continuous $\text{S}{\text{p}}_{{\text{O}}_{2}}$ time traces for a wider range of values compared with the conventional method when validated against the ground truth arterial blood gas $\text{S}{\text{a}}_{{\text{O}}_{2}}$. As such, this method of $\text{S}{\text{p}}_{{\text{O}}_{2}}$ extraction was used in the current study. $\text{S}{\text{p}}_{{\text{O}}_{2}}$ was extracted for the channel with the shallowest skin-cortical tissue path for each dive trial. A 5-s moving average was applied to the extracted $\text{S}{\text{p}}_{{\text{O}}_{2}}$ time traces to remove some signal noise and improve data visualization.

### Statistical Analysis

The differences in minimum heart rate between dive depths and the differences in arterial blood gas and hematology values (${\text{Pa}}_{{\text{O}}_{2}}$, ${\text{Pa}}_{{\text{CO}}_{2}}$, $\text{S}{\text{a}}_{{\text{O}}_{2}}$, and Hb) between dive depths and within arterial blood sampling points of each dive were tested for significance. In addition, the differences in all NIRS-derived metrics ([ΔO_2_Hb], [ΔHHb], [ΔtHb], heart rate, $\text{S}{\text{p}}_{{\text{O}}_{2}}$, and TSI) between start, bottom, ascent, and end times of the dives were tested for significance. For cerebral hemodynamic changes ([ΔO_2_Hb], [ΔHHb], and [ΔtHb]), specifically, the difference in relative changes between dive stages as opposed to the difference in absolute values at dive stages was tested for significance. The start of ascent was calculated by subtracting 15 s and 42 s from the end of the 15-m and 42-m dives, respectively, given that the rate of ascent was 1 m/s. However, this provided an estimate of the start of ascent since the amount of time it took to take the end-dive blood draw was not accounted for. Similarly, the time when the diver reached the bottom of their depth was estimated using the rate of descent (1 m/s) for the dive trials that had missing bottom times (15 m dive for *subject 1* and both depths for *subjects 5* and *6*).

The Shapiro–Wilk test was used to test normality in the distribution of measurement differences due to its higher statistical power with sample sizes smaller than *n* = 50. For comparisons with a normal distribution of measurement differences, a paired samples *t* test was used for significance testing since paired measurements were obtained from the same subjects. For comparisons with a nonnormal distribution of measurement differences, the Wilcoxon signed**-**rank test was used as the nonparametric counterpart of the paired samples *t* test. The significance level for differences was set to *P* < 0.05.

## RESULTS

**Figure 3.**
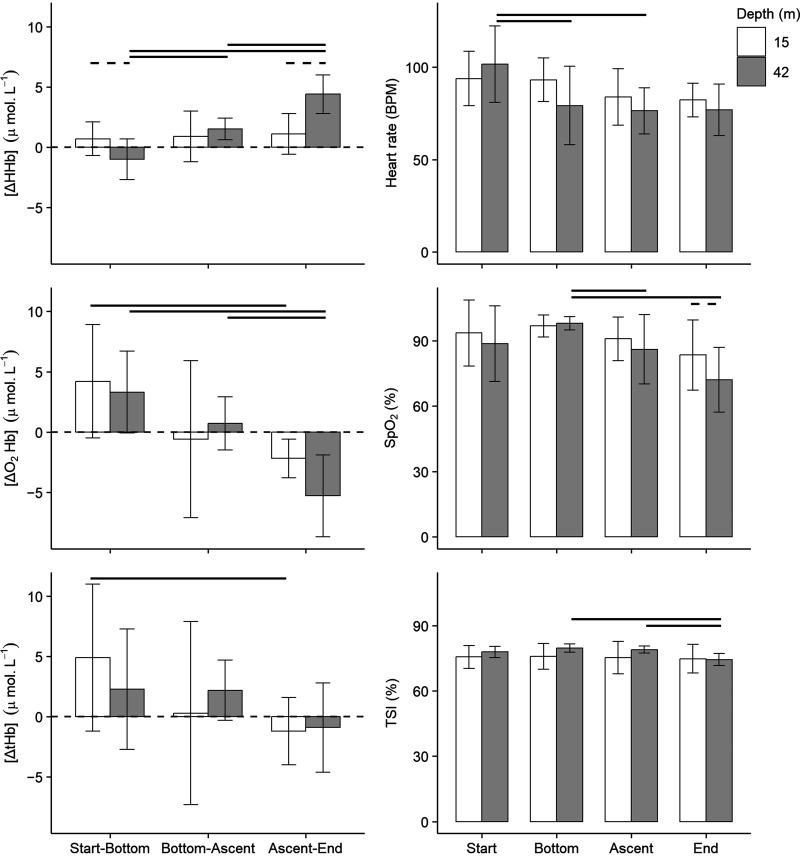
The mean relative changes in [ΔHHb] (μmol/L), [ΔO_2_Hb] (μmol/L), and [ΔtHb] (μmol/L) between the start-bottom, bottom-ascent, and ascent-end of 15- and 42-m dives and mean absolute values of heart rate (beats/min), $\text{S}{\text{p}}_{{\text{O}}_{2}}$ (%), and TSI (%) at the start, bottom, ascent, and end of 15-m and 42-m dives (*n* = 6). Error bars indicate standard deviation. Bold horizontal lines indicate significant differences between stages of the same dive depth, and dashed horizontal lines indicate significant differences in stages between dive depths (*P* < 0.05). For the test statistic and corresponding *P* values for the Shapiro–Wilk test for normality and tests of statistical significance, see Supplemental Tables S2–S4. BPM, beats/min; [ΔHHb], change in concentration of deoxygenated hemoglobin; [ΔO_2_Hb], change in concentration of oxygenated hemoglobin; [ΔtHb], change in concentration of total hemoglobin; TSI, tissue saturation index; $\text{S}{\text{p}}_{{\text{O}}_{2}}$, arterial oxygen saturation.

Continuous NIRS monitoring and arterial blood sampling were successfully completed for all six freedivers. All freedivers recovered successfully from their individual dives and after completion of the dive protocol. One subject (*subject 6*) completed the 42-m dive before the 15-m dive but recovered adequately at the surface in between the two dive depths. The 15-m dives ranged in duration from 45 to 116 s (mean = 80.2 ± 27.2 s) and the 42-m dives ranged in duration from 91 to 140 s (mean = 115.2 ± 16.9 s). The time spent at the bottom of the dive depth ranged from 12 to 59 s (mean = 31.2 ± 24.6 s) for *n* = 3 of the 15-m dives and 12 to 51 s (mean = 34.2 ± 16.4 s) for *n* = 4 of the 42-m dives for which bottom times were collected. All divers, excluding *subject 3*, experienced involuntary breathing movements (IBMs) during ascent from 42 m, and *subject 2* experienced one IBM during ascent from 15 m. Continuous time traces for cerebral hemodynamics ([ΔHHb], [ΔO_2_Hb], and [ΔtHb]), cerebral blood oxygen saturation (TSI), heart rate, and $\text{S}{\text{p}}_{{\text{O}}_{2}}$ are presented for each dive trial in Supplemental Figs. S1–S6. The mean relative changes in cerebral hemodynamic variables between stages of the dives and mean absolute values of TSI, heart rate, and $\text{S}{\text{p}}_{{\text{O}}_{2}}$ at stages of the dives are presented in Table 2 and Fig. 3. The following results are presented in the context of each aim.

**Table 2.** Mean relative changes in cerebral hemodynamic variables between stages of the dives and mean absolute values of TSI, heart rate, and $\text{S}{\text{p}}_{{\text{O}}_{2}}$ at stages of the dives

Depth, m	Mean Relative Changes			Mean Absolute Values			
	Start-bottom	Bottom-ascent	Ascent-end	Start	Bottom	Ascent	End
	*[ΔHHb], μmol/L*			*Heart rate, beats/min*			
15	0.7 ± 1.4	0.9 ± 2.1	1.1 ± 1.7	93.9 ± 14.8	93.2 ± 11.8	83.9 ± 15.2	82.3 ± 9.1
42	−1.0 ± 1.7	1.5 ± 0.9	4.4 ± 1.6	101.7 ± 20.6	79.3 ± 21.2	76.4 ± 12.4	76.9 ± 13.1
	*[ΔO_2_Hb], μmol/L*			$Sp_{O_{2}}$ *, %*			
15	4.2 ± 4.7	−0.6 ± 6.5	−2.2 ± 1.6	93.6 ± 15.2	96.8 ± 5.0	90.9 ± 10.0	83.5 ± 16.1
42	3.3 ± 3.4	0.7 ± 2.2	−5.3 ± 3.4	88.7 ± 17.3	98 ± 3.1	86.1 ± 16.0	72.1 ± 14.9
	*[ΔtHb], μmol/L*			*TSI, %*			
15	4.9 ± 6.1	0.3 ± 7.6	−1.2 ± 2.8	75.6 ± 5.3	75.9 ± 5.9	75.3 ± 7.5	74.8 ± 6.6
42	2.3 ± 5.0	2.2 ± 2.5	−0.9 ± 3.7	77.9 ± 2.6	79.6 ± 1.9	79.0 ± 1.6	74.4 ± 2.7

Values are means ± SD for relative changes in [ΔHHb] (μmol/L), [ΔO_2_Hb] (μmol/L), and [ΔtHb] (μmol/L) between the start-bottom, bottom-ascent, and ascent-end of 15- and 42-m dives, and means ± SD for absolute values of heart rate (beats/min), $\text{S}{\text{p}}_{{\text{O}}_{2}}$ (%), and TSI (%) at the start, bottom, ascent, and end of 15- and 42-m dives (*n* = 6). [ΔHHb], change in concentration of deoxygenated hemoglobin; [ΔO_2_Hb], change in concentration of oxygenated hemoglobin; [ΔtHb], change in concentration of total hemoglobin; TSI, tissue saturation index; $\text{S}{\text{p}}_{{\text{O}}_{2}}$, arterial oxygen saturation.

### Aim 1: Depth-Dependent Effects on Cerebral Hemodynamic and Oxygenation Changes

To assess depth-dependent cerebral hemodynamic changes during breath-hold diving in the absence of exercise, we obtained continuous local hemodynamic measurements of [ΔHHb], [ΔO_2_Hb], and [ΔtHb] and extracted TSI as a local measure of oxygen saturation in the brain.

During descent (start-bottom), there was a marginal increase in [ΔHHb] for 15-m dives and a decrease in [ΔHHb] for 42-m dives (Table 2 and Fig. 3). There was a significant difference in [ΔHHb] between 15-m and 42-m dives during descent (Shapiro–Wilk *W* = 0.898, *P* = 0.363; paired samples *t* test *t*(5) = −2.954, *P* = 0.032). [ΔO_2_Hb] and [ΔtHb] increased during descent for both dive depths, although 15-m dives showed a greater increase than 42-m dives for both variables. From the bottom to the start of ascent (bottom-ascent), divers were resting at the bottom of their dive depth while an arterial blood sample was being drawn. The results for [ΔHHb], [ΔO_2_Hb], and [ΔtHb] were variable at this stage, with variability being captured in the standard deviation for variables of the 15-m dives especially. Nonetheless, there was a significant difference between the change in [ΔHHb] during descent and time spent at the bottom for 42-m dives (Shapiro–Wilk *W* = 0.973, *P* = 0.909; paired samples *t* test *t*(5) = 3.909, *P* = 0.011).

During ascent (ascent-end) [ΔHHb] increased for both depths, although there was a greater increase in [ΔHHb] for 42 m at this stage (Table 2 and Fig. 3). As for descent, there was a significant difference in [ΔHHb] between 15-m and 42-m dives during ascent (Shapiro–Wilk *W* = 0.675, *P* = 0.003, Wilcoxon signed-rank test *V* = 21.000, *P* = 0.031). In addition, there was a significant difference in the change in [ΔHHb] between descent and ascent (Shapiro–Wilk *W* = 0.908, *P* = 0.421; paired samples *t*-test *t*(5) = 6.439, *P* = 0.001), and time spent at the bottom (bottom-ascent) and ascent for 42-m dives (Shapiro–Wilk *W* = 0.979, *P* = 0.945; paired samples *t* test *t*(5) = 8.261, *P* = <0.001). [ΔO_2_Hb] and [ΔtHb] decreased during ascent for both dive depths. There was a significant difference in the change in [ΔO_2_Hb] between descent and ascent of 15 m (Shapiro–Wilk *W* = 0.938, *P* = 0.642; paired samples *t* test *t*(5) = −3.631, *P* = 0.015) and 42-m dives (Shapiro–Wilk *W* = 0.927, *P* = 0.555; paired samples *t* test *t*(5) = −3.341, *P* = 0.021). In addition, there was a significant difference in the change in [ΔO_2_Hb] during the time spent at the bottom and ascent of 42-m dives (Shapiro–Wilk *W* = 0.892, *P* = 0.329; paired samples *t* test *t*(5) = −4.010, *P* = 0.010). [ΔtHb] changed significantly from descent to ascent of 15-m dives (Shapiro–Wilk *W* = 0.954, *P* = 0.774; paired samples *t* test *t*(5) = −2.632, *P* = 0.046).

TSI remained relatively stable within 74.4%–79.6%, showing a marginal increase during descent and decrease during ascent for both dive depths (Table 2 and Fig. 3). There was a significant difference in TSI between the bottom and end (Shapiro–Wilk *W* = 0.890, *P* = 0.321; paired samples *t* test *t*(5) = −3.504, *P* = 0.017) and between the start of ascent and end of 42-m dives (Shapiro–Wilk *W* = 0.910, *P* = 0.434; paired samples *t* test *t*(5) = −4.456, *P* = 0.007).

### Aim 2: Depth-Dependent Effects on Arterial Blood Oxygen Saturation

To investigate the depth-dependent changes in blood oxygen saturation during breath-hold diving, continuous systemic measurements of $\text{S}{\text{p}}_{{\text{O}}_{2}}$ were obtained, along with cross-sectional ground truth arterial blood gas.

#### Arterial oxygen saturation.

**Figure 4.**
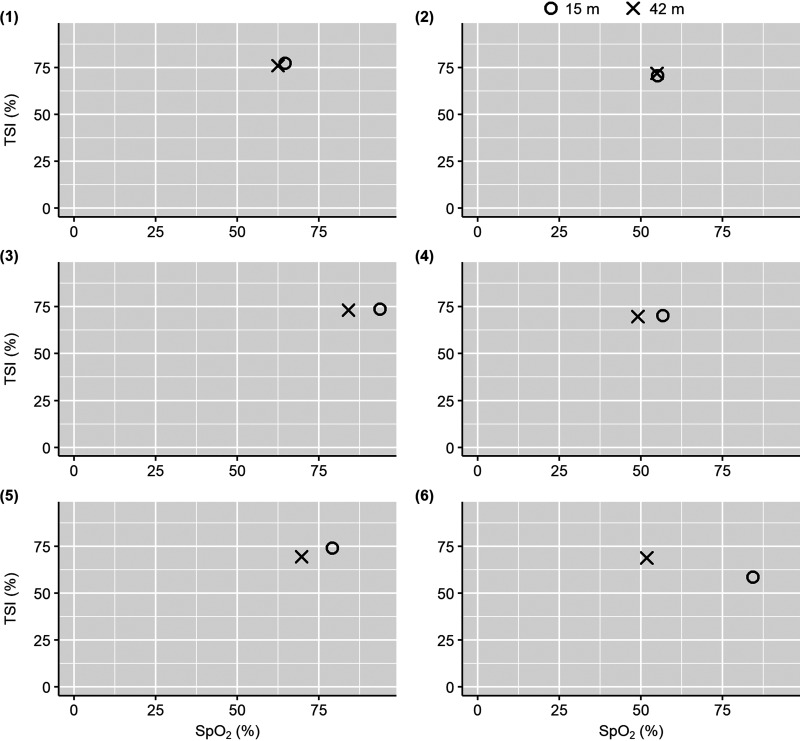
The minimum TSI (%) compared with the minimum $\text{S}{\text{p}}_{{\text{O}}_{2}}$ (%) in 15-m and 42-m dive trials of the subjects (1–6) included in the analysis of this study. TSI, tissue saturation index; $\text{S}{\text{p}}_{{\text{O}}_{2}}$, arterial oxygen saturation.

$\text{S}{\text{p}}_{{\text{O}}_{2}}$ was successfully extracted for all of the 42-m dive trials and all but one of the 15-m dive trials using the self-calibrated $\text{S}{\text{p}}_{{\text{O}}_{2}}$ extraction algorithm. For the 15- m dive of *subject 3*, the self-calibrated algorithm produced an unchanging $\text{S}{\text{p}}_{{\text{O}}_{2}}$ time trace at 100%. The reasons for this anomalous result, whether related to an error in data acquisition or processing, are unknown. As such, the $\text{S}{\text{p}}_{{\text{O}}_{2}}$ time trace for this 15-m dive was extracted using the conventional $\text{S}{\text{p}}_{{\text{O}}_{2}}$ extraction method instead (17). As for all the other dives, the $\text{S}{\text{p}}_{{\text{O}}_{2}}$ time trace for the 15-m dive of *subject 3* was extracted for the shallowest channel, and a 5-s moving average was applied to the extracted $\text{S}{\text{p}}_{{\text{O}}_{2}}$ time trace (Supplemental Fig. S3*B*). However, it is important to note that the conventional method likely yielded a poor estimate of $\text{S}{\text{p}}_{{\text{O}}_{2}}$, given the poor signal quality for this 15-m dive trial. In addition, the last 37 s of the 15-m dive for *subject 6* consisted of noisy signal, which was removed for the comparison of minimum TSI and $\text{S}{\text{p}}_{{\text{O}}_{2}}$ in Fig. 4. The section of the data that was removed for the creation of Fig. 4 is visually presented in Supplemental Fig. S6.

There was an increase in $\text{S}{\text{p}}_{{\text{O}}_{2}}$ during descent to both dive depths. From the end of descent (bottom) to the start of ascent, $\text{S}{\text{p}}_{{\text{O}}_{2}}$ decreased for both dive depths and continued to decrease until the end of the dives for both depths (Table 2 and Fig. 3). There was a significant difference in $\text{S}{\text{p}}_{{\text{O}}_{2}}$ at the end of descent and the end of ascent (Shapiro–Wilk *W* = 0.969, *P* = 0.887; paired samples *t* test *t*(5) = −3.901, *P* = 0.011) and between the end of descent and the start of ascent (i.e., time spent at the bottom) for 42-m dives (Shapiro–Wilk *W* = 0.787, *P* = 0.045, Wilcoxon signed-rank test *V* = <0.001, *P* = 0.031). In addition, there was a significant difference between $\text{S}{\text{p}}_{{\text{O}}_{2}}$ at the end of 15-m and 42-m dives (Shapiro–Wilk *W* = 0.921, *P* = 0.511; paired samples *t* test *t*(5) = −4.183, *P* = 0.009). The changes in $\text{S}{\text{p}}_{{\text{O}}_{2}}$ between dive stages were generally greater in magnitude for 42-m dives than for 15-m dives. The changes in $\text{S}{\text{p}}_{{\text{O}}_{2}}$ and TSI between dive stages followed the same trend, although $\text{S}{\text{p}}_{{\text{O}}_{2}}$ showed a greater magnitude of change overall. In Supplemental Figs. S1–S6, the continuous time traces show that the decrease in $\text{S}{\text{p}}_{{\text{O}}_{2}}$ during ascent closely followed the temporal trend of decreasing TSI, with most subjects showing a greater decrease in $\text{S}{\text{p}}_{{\text{O}}_{2}}$ and TSI during the 42-m dives compared with the 15-m dives (Fig. 4). $\text{S}{\text{p}}_{{\text{O}}_{2}}$ also generally increased at the same point as TSI following the end of a dive.

#### Arterial blood gas.

The arterial blood gas analysis results for ${\text{Pa}}_{{\text{O}}_{2}}$, ${\text{Pa}}_{{\text{CO}}_{2}}$, and $\text{S}{\text{a}}_{{\text{O}}_{2}}$ are presented in Table 3. During descent to 15 m (*point B* to *point C*), mean ${\text{Pa}}_{{\text{O}}_{2}}$ and ${\text{Pa}}_{{\text{CO}}_{2}}$ increased, whereas $\text{S}{\text{a}}_{{\text{O}}_{2}}$ remained close to 100%. At the end of ascent from both dive depths (*points D* and *G*), mean ${\text{Pa}}_{{\text{O}}_{2}}$, ${\text{Pa}}_{{\text{CO}}_{2}}$, and $\text{S}{\text{a}}_{{\text{O}}_{2}}$ decreased from their respective values measured at the bottom of the dives (*points C* and *F*). The drop in mean ${\text{Pa}}_{{\text{O}}_{2}}$ and $\text{S}{\text{a}}_{{\text{O}}_{2}}$ at the end of ascent was greater for the 42-m dives than for the 15-m dives, whereas the drop in ${\text{Pa}}_{{\text{CO}}_{2}}$ was comparable between both dive depths.

**Table 3:** ${\text{Pa}}_{{\text{O}}_{2}}$, ${\text{Pa}}_{{\text{CO}}_{2}}$*, and* $\text{S}{\text{a}}_{{\text{O}}_{2}}$ at each arterial blood sampling point for each subject

Variable	*A*	*B*	*C*	*D*	*F*	*G*
*Subject 1*						
${\text{Pa}}_{{\text{CO}}_{2}}$, mmHg	30.5	30.6	49.5	42.2	34.9	31.3
${\text{Pa}}_{{\text{O}}_{2}}$, mmHg	88.2	119.6	106.5	48.6	214.6	35.4
$\text{S}{\text{a}}_{{\text{O}}_{2}}$, %	98	99	97	77	99	61
*Subject 2*						
${\text{Pa}}_{{\text{CO}}_{2}}$, mmHg	30.8	20.3	33.3	32.8	30.4	25.4
${\text{Pa}}_{{\text{O}}_{2}}$, mmHg	123.8	146.8	235.6	63.2	170.6	49.6
$\text{S}{\text{a}}_{{\text{O}}_{2}}$, %	98	99	100	90	100	83
*Subject 3*						
${\text{Pa}}_{{\text{CO}}_{2}}$, mmHg	29.4	21.5	37.9	34.9	37.7	37.5
${\text{Pa}}_{{\text{O}}_{2}}$, mmHg	104.6	135.9	155.4	36.6	325.4	48.4
$\text{S}{\text{a}}_{{\text{O}}_{2}}$, %	99	99	99	62	99	79
*Subject 4*						
${\text{Pa}}_{{\text{CO}}_{2}}$, mmHg	36.8	32.9	40.1	41.1	42.5	39.4
${\text{Pa}}_{{\text{O}}_{2}}$, mmHg	102.3	123.6	110	33.3	116.2	29.1
$\text{S}{\text{a}}_{{\text{O}}_{2}}$, %	98	99	98	52	98	41
*Subject 5*						
${\text{Pa}}_{{\text{CO}}_{2}}$, mmHg	29.2	19.8	35.5	30.5	27.1	27.1
${\text{Pa}}_{{\text{O}}_{2}}$, mmHg	87.9	139.8	194.1	56.3	229.1	40.9
$\text{S}{\text{a}}_{{\text{O}}_{2}}$, %	99	99	99	86	99	68
*Subject 6*						
${\text{Pa}}_{{\text{CO}}_{2}}$, mmHg	34.4	22.7	38.6	36.5	41.7	38.9
${\text{Pa}}_{{\text{O}}_{2}}$, mmHg	102.4	142.6	196.4	52.5	144.2	35.1
$\text{S}{\text{a}}_{{\text{O}}_{2}}$, %	99	100	99	82	100	50
*Means ± SD*						
${\text{Pa}}_{{\text{CO}}_{2}}$, mmHg	31.9 ± 3.1	24.6 ± 5.7	39.2 ± 5.6	36.3 ± 4.6	35.7 ± 6.1	33.3 ± 6.2
${\text{Pa}}_{{\text{O}}_{2}}$, mmHg	101.5 ± 13.2	134.7 ± 10.8	166.3 ± 51.7	48.4 ± 11.5	200.0 ± 74.5	39.8 ± 8.1
$\text{S}{\text{a}}_{{\text{O}}_{2}}$, %	98.5 ± 0.5	99.2 ± 0.4	98.7 ± 1.0	74.8 ± 14.8	99.2 ± 0.8	63.7 ± 16.3

*n* = 6 subjects. Arterial blood sampling points include baseline sample (A), start of 15-m dive (B), bottom of 15-m dive (C), end of 15-m dive (D), bottom of 42-m dive (F), and end of 42 m dive (G). The mean, SD, and range for blood gas variables at each sampling point are additionally presented in Supplemental Table S5. ${\text{Pa}}_{{\text{O}}_{2}}$, arterial partial pressure of oxygen; ${\text{Pa}}_{{\text{CO}}_{2}}$, arterial partial pressure of carbon dioxide; $\text{S}{\text{a}}_{{\text{O}}_{2}}$, ground truth arterial oxygen saturation.

Significance testing was completed for blood gas values at the bottom of the dive depths and at the end of the dive depths as these were the two arterial blood sampling points that were completed for both 15-m and 42-m dives. These results are presented in Table 4. There was no significant difference in ${\text{Pa}}_{{\text{O}}_{2}}$, ${\text{Pa}}_{{\text{CO}}_{2}}$, and $\text{S}{\text{a}}_{{\text{O}}_{2}}$ between the bottom of the 15-m and 42-m dives (Table 4, C-F). There was also no significant difference in ${\text{Pa}}_{{\text{O}}_{2}}$, ${\text{Pa}}_{{\text{CO}}_{2}}$, and $\text{S}{\text{a}}_{{\text{O}}_{2}}$ between the end of the 15-m and 42-m dives (D-G). Within the 15-m dives, there was no significant difference in ${\text{Pa}}_{{\text{CO}}_{2}}$ between the bottom and the end of the dives (Table 4, C-D). However, $\text{S}{\text{a}}_{{\text{O}}_{2}}$ and ${\text{Pa}}_{{\text{O}}_{2}}$ decreased significantly from the bottom to the end of the 15-m dives. For the 42-m dives, ${\text{Pa}}_{{\text{O}}_{2}}$, ${\text{Pa}}_{{\text{CO}}_{2}}$, and $\text{S}{\text{a}}_{{\text{O}}_{2}}$ decreased significantly from the bottom to the end of the dives (Table 4, *F-G*).

**Table 4.** Test statistic and corresponding P-values for the Shapiro–Wilk test for normality and tests of statistical significance for differences in arterial blood gas values between the bottom and end of each dive depth, and at the bottom and end sampling points between dive depths

Blood Gas Variable	Shapiro–Wilk *W*	*P* Value	Significance Test Statistic	*P* Value
*C-F*				
${\text{Pa}}_{{\text{O}}_{2}}$, mmHg	0.940	0.656	*t* = −0.900	0.409
${\text{Pa}}_{{\text{CO}}_{2}}$, mmHg	0.906	0.413	*t* = 1.222	0.276
$\text{S}{\text{a}}_{{\text{O}}_{2}}$, %	0.701	0.006	*V* = 0.000	0.371
*D-G*				
${\text{Pa}}_{{\text{O}}_{2}}$, mmHg	0.796	0.054	*t* = 1.929	0.112
${\text{Pa}}_{{\text{CO}}_{2}}$, mmHg	0.931	0.585	*t* = 1.399	0.221
$\text{S}{\text{a}}_{{\text{O}}_{2}}$, %	0.933	0.608	*t* = 1.687	0.153
*C-D*				
${\text{Pa}}_{{\text{O}}_{2}}$, mmHg	0.949	0.734	*t* = 6.683	0.001*
${\text{Pa}}_{{\text{CO}}_{2}}$, mmHg	0.984	0.969	*t* = 2.291	0.071
$\text{S}{\text{a}}_{{\text{O}}_{2}}$, %	0.878	0.262	*t* = 4.058	0.010*
*F-G*				
${\text{Pa}}_{{\text{O}}_{2}}$, mmHg	0.920	0.504	*t* = 5.634	0.002*
${\text{Pa}}_{{\text{CO}}_{2}}$, mmHg	0.906	0.409	*t* = 3.044	0.029*
$\text{S}{\text{a}}_{{\text{O}}_{2}}$, %	0.943	0.681	*t* = 5.432	0.003*

The test statistic (df = 5) and corresponding *P* values for the Shapiro–Wilk test for normality and tests of statistical significance for differences in ${\text{Pa}}_{{\text{O}}_{2}}$, ${\text{Pa}}_{{\text{CO}}_{2}}$, and $\text{S}{\text{a}}_{{\text{O}}_{2}}$ between the bottom of the 15- and 42-m dives (C-F), the end of the 15- and 42-m dives (D-G), the bottom and end of the 15-m dives (C-D), and the bottom and end of the 42-m dives (F-G). The test statistic and corresponding *P* value are presented for either the paired samples *t* test (*t*) or the Wilcoxon signed-rank test (*V*) based on whether measurement differences showed a normal or nonnormal distribution. ${\text{Pa}}_{{\text{O}}_{2}}$, arterial partial pressure of oxygen; ${\text{Pa}}_{{\text{CO}}_{2}}$, arterial partial pressure of carbon dioxide; $\text{S}{\text{a}}_{{\text{O}}_{2}}$, ground truth arterial oxygen saturation.

*Significant difference.

Hemoglobin concentration (Hb, g/dL) was included in the arterial blood analysis to assess whether oxygen content may have been maintained through splenic contraction despite the decrease in ${\text{Pa}}_{{\text{O}}_{2}}$ and arterial oxygen saturation during ascent. Given that previous studies reported an increase in Hb at the end of apnea trials compared with baseline (28–30), significance testing was completed for differences in Hb across all arterial blood sampling points (Supplemental Table S6). There was no significant difference in Hb between any of the arterial blood sampling points (Supplemental Table S7).

### Aim 3: Depth-Dependent Effects on Heart Rate

Heart rate data were successfully extracted for each dive trial in the absence of exercise. The extracted heart rate data were variable across both depths (Supplemental Figs. S1–S6). Nonetheless, means ± SD values indicated that heart rate decreased across all stages of the dives for both dive depths (Table 2 and Fig. 3). The changes in heart rate were greater for 42-m dives than for 15-m dives. There was a significant difference in heart rate between the start and end of descent (bottom) (Shapiro–Wilk *W* = 0.986, *P* = 0.975; paired samples *t* test *t*(5) = −2.831, *P* = 0.037) and between the start of descent and start of ascent for 42-m dives (Shapiro–Wilk W = 0.981, *P* = 0.955; paired samples *t* test *t*(5) = −6.278, *P* = 0.002). There was no significant difference between the minimum heart rate for 15-m and 42-m dives (Shapiro–Wilk *W* = 0.928, *P* = 0.562; paired samples *t* test *t*(5) = −1.017, *P* = 0.356).

## DISCUSSION

In the current study, we present a standardized experimental protocol for freediving experiments in which confounding variables of physical exercise and depth-related differences were accounted for through the use of sled-assisted dives and two different depths. We measured continuous cerebral hemodynamic changes using a waterproofed CW-NIRS system and retrospectively extracted TSI and systemic measures of heart rate and $\text{S}{\text{p}}_{{\text{O}}_{2}}$ for six participant divers. The extracted continuous $\text{S}{\text{p}}_{{\text{O}}_{2}}$ time traces were validated using arterial blood gas measurements, which were obtained at various points throughout the diving protocol. The results are discussed in the context of each aim below.

### Aim 1: Depth-Dependent Effects on Cerebral Hemodynamic and Oxygenation Changes

The cerebral hemodynamic changes during the freediving trials were initially characterized by an increase in cerebral oxygenation (TSI and [ΔO_2_Hb]) and cerebral blood volume ([ΔtHb]) early in the dive (Table 2 and Fig. 3). Similarly, another study found an increase in NIRS-derived cerebral blood volume with a concomitant decrease in peripheral blood volume in freedivers diving to 40 m of depth with a monofin (13). These changes are characteristic of the dive response, which couples a decrease in cardiac output with peripheral vasoconstriction to centralize blood flow and preferentially perfuse the brain (31). The significant difference in [ΔHHb] between descent to 15 m and 42 m could be attributed to greater ${\text{Pa}}_{{\text{O}}_{2}}$ at the bottom of 42 m (Table 3) causing a rightward shift in the oxygen-hemoglobin dissociation curve, and thereby a decreased fraction of deoxygenated hemoglobin compared with 15 m. In addition, the concomitant greater increase in [ΔtHb] and [ΔO_2_Hb] during descent to 15 m compared with 42 m could be attributed to a greater reduction in cardiac output for 42-m dives, which is supported by the significant decrease in heart rate during descent to 42 m (Fig. 3).

During ascent, there was a shift in oxygenation characterized by an increase in [ΔHHb], decrease in [ΔO_2_Hb], and decrease in TSI. We suggest that this shift in oxygenation was driven by the concomitant decrease in systemic $\text{S}{\text{p}}_{{\text{O}}_{2}}$ during ascent (see *Aim 2: Depth-Dependent Effects on Arterial Blood Oxygen Saturation*). Mean cerebral blood volume did not increase during ascent in our study but instead showed a marginal decrease for both depths. However, the continuous time traces in Supplemental Figs. S1–S6 show an initial decrease in [ΔtHb] during ascent followed by a sudden increase until the end of the dives for both depths. Given the initial decrease in [ΔtHb], the increasing trend in the latter half of ascent may not have been captured in the mean changes.

The phenomenon of rising cerebral blood volume engendered by increasing [ΔHHb] and a concomitant decline in TSI was observed in longer dives to depth of elite freedivers (12). However, in our study, the hemodynamic and oxygenation trends during descent and ascent were seen at both dive depths (shallow and deep), although the magnitudes of change were generally greater for 42-m dives than for 15-m dives; also reflected in results from significance testing (Fig. 3). Although the dive trials in our study followed a standardized protocol, elite freedivers in the study by McKnight et al. (12) participated in a variety of freediving disciplines, which likely influenced the variability in observed cerebral hemodynamic changes. Future research should investigate the effects of various disciplines on cerebral hemodynamics to elucidate ways in which specific freediving disciplines may aid or detriment dive duration and recovery.

### Aim 2: Depth-Dependent Effects on Arterial Blood Oxygen Saturation

Continuous systemic measurements of arterial blood oxygen saturation delivered to the brain ($\text{S}{\text{p}}_{{\text{O}}_{2}}$) increased during descent for both dive depths (Table 2 and Fig. 3). This is likely due to ambient pressure changes associated with the descent phase. As ambient pressure increases with depth, the lungs compress, thereby increasing arterial ${\text{Pa}}_{{\text{O}}_{2}}$, which maintains the high levels of $\text{S}{\text{p}}_{{\text{O}}_{2}}$ (1, 2, 12). During ascent, $\text{S}{\text{p}}_{{\text{O}}_{2}}$ decreased for both dive depths, although with a greater decrease and statistically significant change in $\text{S}{\text{p}}_{{\text{O}}_{2}}$ during the 42-m dives. Moreover, the ground truth arterial blood gas values of ${\text{Pa}}_{{\text{O}}_{2}}$ and $\text{S}{\text{a}}_{{\text{O}}_{2}}$ confirmed hyperoxia at the bottom of the dives and a significant decrease in arterial oxygenation from the bottom to the end of the dives for both depths (Table 4). The concurrent decline in arterial ${\text{Pa}}_{{\text{O}}_{2}}$ and $\text{S}{\text{p}}_{{\text{O}}_{2}}$ presented during ascent in our study can be attributed to lung reinflation under decreasing ambient pressure. As ambient pressure decreases during ascent from depth, the lungs reinflate, causing arterial blood gas tensions (including ${\text{Pa}}_{{\text{O}}_{2}}$) to decrease (1, 2, 12). For some of the dive trials, there was a delay between the end of apnea and the minimum of $\text{S}{\text{p}}_{{\text{O}}_{2}}$ and TSI. The cause for this delay was likely the circulation time required for oxygenated blood from the lungs to reach cerebral tissue (32).

Although there was a significant difference in mean $\text{S}{\text{p}}_{{\text{O}}_{2}}$ between 15-m and 42-m dives, mean TSI at the end of both dive depths was 74% (Table 2 and Fig. 3). The difference in TSI and $\text{S}{\text{p}}_{{\text{O}}_{2}}$ may be influenced by the mixed arterial-venous contribution to measures of TSI versus the sole arterial contribution to measures of $\text{S}{\text{p}}_{{\text{O}}_{2}}$. Despite a greater decrease in $\text{S}{\text{p}}_{{\text{O}}_{2}}$ during ascent from 42 m, cerebral tissue-specific oxygenation may have been maintained at a similar level by the end of ascent from both depths through a venous contribution. For example, increased cerebral blood flow (CBF) may decrease the arterial-venous oxygen saturation difference (20).

The arterial blood gas results concur with those previously published by Qvist et al., Bosco et al., and Scott et al. (2, 5, 21), in which freedivers performing sled-assisted and unassisted dives were sampled for arterial blood at the bottom of their breath-hold diving depths. All studies reported an increase in ${\text{Pa}}_{{\text{O}}_{2}}$ at the bottom of the dive depths compared with baseline samples and a decrease in ${\text{Pa}}_{{\text{O}}_{2}}$ from the bottom of the dive depths compared with the end of the dives. Bosco et al. (2) also reported that $\text{S}{\text{a}}_{{\text{O}}_{2}}$ was maintained at depth and decreased after ascent. In our study, we report a greater decrease in ${\text{Pa}}_{{\text{O}}_{2}}$ and $\text{S}{\text{a}}_{{\text{O}}_{2}}$ during sled-assisted ascent from 42 m than in the study by Bosco et al. during ascent from 40 m despite a shorter mean dive duration. However, in the study by Bosco et al., the end-dive arterial blood sample was taken within 2 min of surfacing and after the resumption of breathing, whereas end-dive blood samples in our study were taken before the resumption of breathing, while the divers’ heads were still submerged (2). Although these arterial blood gas results have further evidenced the effect of lung compression and expansion on arterial blood gas tensions during freediving to depth, our NIRS-derived $\text{S}{\text{p}}_{{\text{O}}_{2}}$ traces provide continuous measurements of arterial oxygenation, which remain elusive in human deep-diving studies.

The changes in $\text{S}{\text{p}}_{{\text{O}}_{2}}$ described in this study follow those presented in the study by McKnight et al. (12), in which $\text{S}{\text{p}}_{{\text{O}}_{2}}$ was also extracted from NIRS measurements obtained during freediving trials but using the conventional $\text{S}{\text{p}}_{{\text{O}}_{2}}$ extraction algorithm validated against pulse oximetry measures of $\text{S}{\text{p}}_{{\text{O}}_{2}}$ (17). The current study is the first on actively diving humans to apply and validate an $\text{S}{\text{p}}_{{\text{O}}_{2}}$ extraction algorithm that accounts for changes in optical pathlength, which otherwise introduce significant error to $\text{S}{\text{p}}_{{\text{O}}_{2}}$ estimates (33, 34). We, therefore, reason that the trends in $\text{S}{\text{p}}_{{\text{O}}_{2}}$ presented in the current study likely reflect the most accurate dynamics of continuous arterial blood oxygen changes in breath-hold diving humans. In addition, we encourage significant caution in the interpretation of existing $\text{S}{\text{p}}_{{\text{O}}_{2}}$ estimates generated in breath-hold diving studies using conventional pulse oximetry. Although the NIRS-derived $\text{S}{\text{p}}_{{\text{O}}_{2}}$ estimates presented in the current study were validated using ground truth $\text{S}{\text{a}}_{{\text{O}}_{2}}$ measures obtained from arterial blood gas analysis, there were some discrepancies between the two measures with regard to the results from significance testing. Increased usage and improvement of the $\text{S}{\text{p}}_{{\text{O}}_{2}}$ extraction algorithm developed by Wu et al. (18) could reduce the need for invasive arterial blood sampling techniques to obtain reliable data in future studies.

### Aim 3: Depth-Dependent Effects on Heart Rate

Increased heart rate during ascent has been previously reported as a result of active swimming to overcome the effect of negative buoyancy at depth (8, 9). In our study, divers descended and ascended using the assistance of a sled so that the effect of exercise on heart rate was removed. The changes in heart rate across dive trials were variable (Supplemental Fig. S1*B*, Supplemental Fig. S2*B*, Supplemental Fig. S3*B*, Supplemental Fig. S4*B*, Supplemental Fig. S5*B*, and Supplemental Fig. S6*B*), which can be partly attributed to individual variability, given differences in the level of apneic training (35–37), age (38, 39), and mental state throughout the dive trials. In a study by Mulder et al. (11) using pulse oximetry measures of heart rate, authors also reported variable heart rate across shallow and deep (mean of 17 msw and 53 msw, respectively) constant weight and free immersion dives. However, the variability in heart rate in this study was attributed to the “autonomic conflict” of parasympathetically induced bradycardia and sympathetically activated exercise tachycardia, which may result in cardiac arrhythmias (11). In our study, we found no significant difference between the minimum heart rate of 15-m and 42-m dives, suggesting that the observed cardiovascular changes did not differ significantly between the dives to both depths. Similarly, Mulder et al. (11) found that minimum heart rate did not differ between shallow and deep constant weight or free immersion dives and reported individual variation in the temporal occurrence of minimal heart rate throughout the dives. The significant decrease in heart rate during descent to 42 m in our study was likely a result of the dive response, which elicits bradycardia upon submersion to maintain arterial blood pressure during peripheral vasoconstriction (31).

Importantly, the NIRS-derived hemodynamic data, which were used to extract heart rate, were subject to some noise, which may have affected the accuracy of the time traces in heart rate. The methods used for heart rate and $\text{S}{\text{p}}_{{\text{O}}_{2}}$ extraction rely on clean signal from the NIRS measurements without confounding artefacts. In future studies, freedivers could be equipped with an accelerometry tag to identify and filter movement artifacts that may contaminate the NIRS measurements (12).

### Further Arterial Blood Gas Changes

As previously mentioned, the arterial blood gas changes throughout stages of the dives to each depth can largely be attributed to ambient pressure changes, which has been demonstrated in previous studies (2–6). The greater magnitude of change in ${\text{Pa}}_{{\text{O}}_{2}}$ than ${\text{Pa}}_{{\text{CO}}_{2}}$ over the course of the dive trials can be attributed to the human body’s buffering capacity for carbon dioxide due to its greater solubility in tissues and blood compared with oxygen (2–6, 40). The lower degree of ambient pressure changes experienced in 15-m dives compared with 42-m dives may also have contributed to the change in mean ${\text{Pa}}_{{\text{CO}}_{2}}$ during ascent from 15 m being insignificant (Table 4). In addition, the shorter duration of 15-m dives could be associated with a lower metabolic production of carbon dioxide, which also may have contributed to the limited ${\text{Pa}}_{{\text{CO}}_{2}}$ changes during ascent from 15 m compared with 42 m.

The decrease in mean ${\text{Pa}}_{{\text{CO}}_{2}}$ and increase in mean ${\text{Pa}}_{{\text{O}}_{2}}$ from the baseline sampling point to the start of the 15 m dives (Table 3) were likely a result of the predive breathing preparation. Although subjects followed a controlled preparatory breathing routine and were instructed not to hyperventilate, our results showed hypocapnia before descent to 15 m (24.6 ± 5.7; means ± SD) compared with resting conditions, which would indicate hyperventilation. Similarly, in another study by Scott et al. (5), ${\text{Pa}}_{{\text{CO}}_{2}}$ fell to 21.0 mmHg following deep breathing and glossopharyngeal insufflation of a freediver’s preparatory breathing, indicating hyperventilation. Given that the controlled preparatory breathing was routine for the subjects in our study, the reported hypocapnia can be considered a “normal” prediving condition for these freedivers.

There was no significant difference in ${\text{Pa}}_{{\text{O}}_{2}}$, ${\text{Pa}}_{{\text{CO}}_{2}}$, and $\text{S}{\text{a}}_{{\text{O}}_{2}}$ at the bottom and end sampling points between the 15-m and 42-m dives (Table 4), which further supports that the observed hemodynamic and systemic changes were occurring at both depths. In addition, these findings suggest that the difference in dive duration between the two depths was not great enough to cause a significant difference in ${\text{Pa}}_{{\text{O}}_{2}}$ and $\text{S}{\text{a}}_{{\text{O}}_{2}}$ at the end-dive sampling point. However, caution should be taken in the interpretation of these results given the small sample size. For instance, the difference in $\text{S}{\text{a}}_{{\text{O}}_{2}}$ between the end of 15-m and 42-m dives varied from 7% (*subject 2*) to 32% (*subject 6*), showing a large range potentially influenced by differences in the subjects’ freediving experience and training intensity or measurement error.

### Depth-Dependent Effects on Cardiovascular and Blood Oxygen Regulation

The combination of continuous local hemodynamic and oxygenation measurements in the brain ([ΔHHb], [ΔO_2_Hb], [ΔtHb], and TSI) with continuous systemic measurements of arterial blood oxygen saturation delivered to the brain ($\text{S}{\text{p}}_{{\text{O}}_{2}}$) and heart rate can help disentangle depth-dependent effects on cardiovascular and blood oxygen regulation. Dive duration and barometric pressure changes are two main depth-dependent drivers of physiological changes associated with freediving (1). Prolonged apnea causes greater cerebral deoxygenation, in part, due to increased oxygen consumption over time (1, 2, 4, 6). In our study we found an increased drop in NIRS-derived and arterial blood gas measures of oxygenation (TSI, $\text{S}{\text{p}}_{{\text{O}}_{2}}$, ${\text{Pa}}_{{\text{O}}_{2}}$, and $\text{S}{\text{a}}_{{\text{O}}_{2}}$) during 42-m dives compared with 15-m dives. Given that the 42-m dives were on average longer than the 15-m dives, it is likely that increased metabolic oxygen consumption over longer dive durations contributed to the increased cerebral and systemic deoxygenation in 42-m dives compared with 15-m dives.

The physiological changes experienced during freediving are also influenced by the previously discussed barometric pressure changes associated with depth. McKnight et al. (12) found an inverse relationship between heart rate and depth, which suggests that depth-induced intrathoracic pressure changes may have an isolated effect on heart rate in addition to bradycardia induced by the diving response. However, these results were confounded by different levels of exercise during diving disciplines. In another study by Lemaître et al. (10), ECG-recorded heart rate abruptly increased in the last 20–25 m of ascent from 70 m, after most of the muscular effort of ascent in freedivers completing constant-weight dives. The authors of this study attributed the slower relative increase in heart rate during stages of ascent when muscular effort was heightened to the dive response overriding exercise tachycardia (10). Given that the greatest relative increase in barometric pressure occurs at ∼10 m of depth compared with sea level, the abrupt increase in heart rate within the last 20–25 m of ascent could, in part, be attributed to the greater relative increase in intrathoracic pressure at this stage of ascent. Although the effect of exercise was accounted for in our study, we could not confirm whether ambient pressure changes alone significantly impacted heart rate. Equally, given that there was no overall increase in heart rate during ascent from either depth, we could not conclude that increased cardiac output may have contributed to cerebral deoxygenation during ascent.

The prominent driver of the cerebral hemodynamic changes during ascent from both depths was likely decreasing $\text{S}{\text{p}}_{{\text{O}}_{2}}$ due to lung reinflation under decreasing ambient pressure (41). The decrease in TSI during ascent temporally aligned with the decrease in $\text{S}{\text{p}}_{{\text{O}}_{2}}$ that was observed for the majority of the dive trials including both dive depths (Supplemental Figs. S1–S6). In addition, the greater decrease in $\text{S}{\text{p}}_{{\text{O}}_{2}}$ during 42-m dives coincided with a greater, although not significant, decrease in TSI compared with 15-m dives for most subjects (Figs. 3 and 4). The decrease in $\text{S}{\text{p}}_{{\text{O}}_{2}}$ during ascent could have stimulated a compensatory increase in cerebral blood flow (CBF) to maintain oxygen delivery to the brain, a well-documented response to hypoxic stimuli in humans (42). Although CBF is regulated by several interacting mechanisms, deoxygenated hemoglobin is the primary regulator for increasing CBF under hypoxia (42, 43). Therefore, increasing [ΔHHb] as a result of decreasing $\text{S}{\text{p}}_{{\text{O}}_{2}}$ and ${\text{Pa}}_{{\text{O}}_{2}}$ during ascent could have stimulated cerebral vasodilation to maintain cerebral oxygen delivery, which would explain the concomitant increase in cerebral blood volume. However, as seen in patients with obstructive sleep apnea with the same cerebral hemodynamic and $\text{S}{\text{p}}_{{\text{O}}_{2}}$ changes, the increase in CBF could not compensate for arterial deoxygenation, causing TSI to continue decreasing (44–48).

Another potential contributor to the cerebral hemodynamic changes during ascent could be IBMs. The increasingly negative inspiratory intrathoracic pressure swings from IBMs have been found to cause an increase in left ventricular stroke volume, which in turn increases CBF (49, 50). In addition to increasing CBF, IBMs may also have inhibited cerebral venous outflow. IBMs resemble repeated Müller maneuvers in which inspiratory effort is attempted against a closed glottis. Negative intrathoracic pressure produced by the Müller maneuver causes the internal jugular vein to collapse at the thoracic inlet, decreasing blood flow through the superior vena cava and thereby impeding cerebral venous return to the right heart (51). The same mechanism could engender cerebral venous congestion during IBMs, contributing to the simultaneous rise in [ΔHHb] and [ΔtHb] during the latter half of ascent. However, it is unknown whether repeated individual short-duration IBMs would elicit the same overall effect as a sustained Müller maneuver. In the current study, all but one diver experienced IBMs during ascent from 42 m, and only one diver experienced an IBM during ascent from 15 m. Therefore, it is unlikely that IBMs were the main driver of the cerebral hemodynamic changes during ascent, which were observed for both dive depths. However, they may have contributed to the greater magnitude of changes observed during ascent from 42 m compared with 15 m.

Trained freedivers present extreme hypoxic tolerance, in part due to higher cerebrovascular reactivity sustaining cerebral oxygen delivery and maintaining cerebral metabolism (52–54), which allows them to tolerate $\text{S}{\text{a}}_{{\text{O}}_{2}}$ tensions down to 41% as observed in this study (*subject 4*, Table 3). However, the changing arterial blood gas tensions associated with freediving to depth still pose a pathophysiological threat. The effect of pressure on arterial blood gas tensions has been linked to hypoxic syncope during ascent, or shallow-water blackout, which is a loss of consciousness as a result of the decreasing barometric pressure during ascent, causing blood oxygen levels that supply the brain to decline (1–3, 6, 7). The use of broadband NIRS to measure relative changes in concentration of cytochrome-c-oxidase could supplement the methods of our study in the future to assess at what stage cerebral deoxygenation during ascent may inhibit cerebral metabolic function (55). In addition, diffuse correlation spectroscopy can measure CBF and, if modified for use underwater, could help elucidate to what extent CBF is contributing to increasing cerebral blood volume ([ΔtHb]) during ascent (56).

### Limitations and Methodological Considerations

The CW-NIRS system used in this study consisted of one sensor head measuring cerebrovascular changes at the prefrontal cortex. However, the hemodynamic variables ([ΔHHb], [ΔO_2_Hb], and [ΔtHb]) presented magnitudes of change up to 17 μmol/L, which are representative of global cerebral changes as opposed to regional cortical changes which occur at ≤ 1 μmol/L (57). Systemic measures of heart rate and $\text{S}{\text{p}}_{{\text{O}}_{2}}$ do not present regional differences. Nonetheless, the use of multiple sensor heads would confirm whether the observed changes in cerebral hemodynamic and oxygenation measures of [ΔHHb], [ΔO_2_Hb], [ΔtHb], and TSI were representative of cerebrovascular changes in the whole brain. TSI estimates tissue-specific oxygen saturation with mixed arterial-venous contribution, whereas $\text{S}{\text{p}}_{{\text{O}}_{2}}$ is specific to arterial changes in oxygen saturation, an important consideration when comparing these two metrics.

The analyses for this study were limited by the small sample size (*n* = 6) due to the reliance on volunteers who were experienced in freediving and comfortable with arterial cannulation and submerged phlebotomy. To ensure the safety of the divers, the recovery period durations in between the two dives of the diving protocol were established by the individuals. We found no significant difference in NIRS-derived measures of heart rate, $\text{S}{\text{p}}_{{\text{O}}_{2}}$, and TSI at the start of 15-m and 42-m dives, suggesting that the self-determined time between dives to each depth provided an adequate recovery for participant divers. Still, the lack of standardization of this recovery period may have influenced some of the systemic and cerebral hemodynamic and oxygenation changes of the 42-m dives differentially between subjects. In addition, there was no arterial blood sample for the beginning of the 42-m dives to limit the amount of blood drawn from participant divers. Although divers were asked to complete the same predive breathing preparation for both dives, the effects from the previous 15-m dive and the lack of standardization for the recovery period could have influenced the arterial blood gas values at the beginning of the 42-m dives, in which case we cannot assume that the arterial blood gas values are identical at the start of 15-m and 42-m dives. In the future, dives to each depth could be completed on separate days so that blood can be drawn at each stage of a dive regardless of the dive depth.

Physiological changes resulting from IBMs were discussed as potential contributors to the observed cerebral hemodynamic changes during ascent. The effect of individual and several consecutive IBMs on the cerebral hemodynamic changes could be better interpreted by verifying their temporal occurrence through the use of a pneumatic respiratory belt to monitor chest expansion. Then, in a separate protocol, the influence of IBMs on the integrity of systemic venous trunks in the thoracic inlet could be studied by imaging the internal jugular vein and superior vena cava during IBMs of maximal breath-holds using pulsed Doppler echocardiography in dry conditions (51). This protocol could clarify whether cerebral venous congestion caused by the Müller maneuver is also engendered by IBMs.

### Perspectives and Significance

In this study, we instrumented experienced freedivers with a continuous-wave near-infrared spectroscopy (NIRS) device, which provided continuous local hemodynamic and oxygenation measurements in the brain and systemic measures of heart rate and $\text{S}{\text{p}}_{{\text{O}}_{2}}$. Participant divers performed two sled-assisted breath-hold dives, one to 15 m and one to 42 m of depth, thereby accounting for the confounding variables of exercise and depth during human breath-hold diving. By obtaining ground truth arterial blood gas values, we also validated extracted $\text{S}{\text{p}}_{{\text{O}}_{2}}$ measurements and the interpretation of changes observed in NIRS-derived hemodynamic signals. We aimed to investigate the depth-dependent effects on *1*) cerebral hemodynamic and oxygenation changes, *2*) $\text{S}{\text{p}}_{{\text{O}}_{2}}$, and *3*) heart rate during breath-hold diving in the absence of exercise. The observed changes in both cerebral hemodynamic and systemic variables during descent to each depth corresponded to the expected physiological changes of the dive response. In addition, arterial blood gas results confirmed compression hyperoxia due to increased pressure at depth for both dive depths. During ascent from both depths, there was an increase in cerebral blood volume and a decrease in cerebral oxygenation, which was likely driven by the concomitant decreasing $\text{S}{\text{p}}_{{\text{O}}_{2}}$, a trend that is indicative of decreasing arterial blood gas tensions associated with decreasing pressure during ascent from depth. Although the observed changes in $\text{S}{\text{p}}_{{\text{O}}_{2}}$ and cerebral hemodynamics and oxygenation occurred during dives to both depths, the changes associated with 42-m dives generally occurred at a greater magnitude compared with 15-m dives. We found no clear trend of increasing heart rate during ascent that could have been attributed to depth-induced intrathoracic pressure changes in the absence of exercise. However, the NIRS-derived hemodynamic data, which were used to extract heart rate, were subject to some noise, which may have affected the accuracy of the time traces in heart rate. We suggest that the protocol presented in the current study be used in future research with the addition of other optical imaging techniques to elucidate the extent to which each of these mechanisms may be contributing to the described cerebral hemodynamic changes. A better understanding of the underlying mechanisms causing cerebral deoxygenation during ascent could help inform routine practices used by freedivers to dampen the risk of hypoxic injury, such as shallow-water blackout.

## DATA AVAILABILITY

Source data for this study are openly available at https://doi.org/10.6084/m9.figshare.23816817.v1.

## SUPPLEMENTAL MATERIAL

10.6084/m9.figshare.25315048 Supplemental Figs. S1–S6 and Supplemental Tables S1–S7: https://doi.org/10.6084/m9.figshare.25315048.

## GRANTS

Funding was received from the Office of Naval Research ONR N00014-21-1-2303 (to J.C.M., J.M.K., and G.B.) and N00014-20-1-2579 (to J.C.M. and G.B.), the National Institutes of Health NIH/NIBIB SBIR R43-EB030625 (to J.M.K.), and the National Science Foundation NSF SBIR 2025901 (to J.M.K.).

## DISCLOSURES

No conflicts of interest, financial or otherwise, are declared by the authors.

## AUTHOR CONTRIBUTIONS

G.B., J.M.K., and J.C.M. conceived and designed research; T.G., G.B. and M.P. performed experiments; E.-M.S.B., A.R., and J.W. analyzed data; E.-M.S.B. interpreted results of experiments; E.-M.S.B. prepared figures; E.-M.S.B. drafted manuscript; E.-M.S.B., T.A.G., G.B., J.M.K., M.P., A.R., J.W., and J.C.M. edited and revised manuscript; E.-M.S.B., T.A.G., G.B., J.M.K., M.P., A.R., J.W., and J.C.M. approved final version of manuscript.
